# Bafilomycin A1 Accelerates Chronic Refractory Wound Healing in db/db Mice

**DOI:** 10.1155/2020/6265701

**Published:** 2020-07-02

**Authors:** Fan Wang, Chao Zhang, Linna Dai, Yulu Zhang, Yongxue Wang, Yongwei Hao, Shenglu Ji, Zhihao Xu, Na Han, Hongli Chen, Qiqing Zhang, Wenbin Nan

**Affiliations:** ^1^Life Science and Health Research Institute, College of Life Science and Technology, Xinxiang Medical University, Xinxiang, 453003 Henan, China; ^2^Tsingtao Brewery No.4, Tsingtao Brewery Co., Ltd., Qingdao, 266041 Shandong, China; ^3^Institute of Biomedical Engineering, Chinese Academy of Medical Sciences, Tianjin 300000, China; ^4^Bote Biotech. Col., Ltd., Fuzhou, 350000 Fujian, China

## Abstract

Numerous studies have reported that autophagy plays an important role in chronic wound healing, and enhancement of autophagic activity impairs cutaneous wound healing. The autophagy inhibitor Bafilomycin A1 (Baf A1) inhibits autophagy by preventing the formation of autophagosomes. This study aimed at elucidating the effect of Bafilomycin A1 on chronic refractory wound healing in diabetic mice. A total of 40 diabetic (db/db) mice and 20 nondiabetic (db/m) mice were used in this study. Full-thickness skin defects were generated in the db/db mice models, which were then divided into the following two groups: the nontreated (db/db group) and Baf A1-treated groups (Baf A1 group). The same skin defects were generated in db/m mice (db/m group) to serve as a control. We demonstrated that Baf A1 treatment significantly accelerated wound healing in db/db mice and exerted good healing effects. Moreover, Baf A1 inhibited autophagy in the newly generated epidermis and had minor effects on metabolism in db/db mice. PCNA expression, as detected by immunohistochemistry, and collagen thickness, as detected by Masson's trichrome staining on the 14th day, were higher in the db/m and Baf A1 groups than in the db/db group. In addition, the expression of the proinflammatory cytokine TNF-*α* in the db/m and Baf A1 groups increased significantly on day 6, and the expression of the anti-inflammatory cytokine IL-10 also increased significantly on day 9. However, there were no significant changes in the expression levels of TNF-*α* and IL-10 in the db/db group. Therefore, Baf A1 may accelerate diabetic chronic refractory wound healing by promoting cell proliferation, collagen production, and regulating the inflammatory balance.

## 1. Introduction

Wound healing is a complex and dynamic process that is affected by many factors. Chronic refractory wounds can be caused by numerous conditions, such as advanced age, poor nutrition, infection, stress, and medication [1–3]. Diabetes mellitus (DM) is one of the most common factors causing chronic wounds, and delayed wound healing is one of the most serious complications of diabetes [4]. The healing of surgical wounds [5], dental extraction sockets [6], and foot ulcers [7] is retarded in diabetic patients. Despite considerable studies on the pathogenesis of delayed wound healing caused by diabetes, the underlying molecular mechanisms are poorly understood.

Autophagy is a fundamental intracellular catabolic process in which autophagosomes deliver cellular components to the lysosome complex for degradation and recycling [8, 9]. Recently, increasing evidence has implicated autophagic dysfunction in the development of neurodegenerative diseases, cancer, infection, and aging [10], and some studies have linked autophagic activity and its regulation with wound healing. For example, a study conducted by Guo et al. showed that advanced glycation end products- (AGEs-) induced autophagy impairs cutaneous wound healing [11], and a study conducted by Zeng et al. indicated that endothelial cell-derived small extracellular vesicles suppress cutaneous wound healing by regulating fibroblast autophagy [12]. Some studies have suggested that the mechanisms promoting wound healing, melatonin production, and the effects of far-infrared therapy are also related to the regulation of autophagy [13–15]. Through these studies, we found that wound healing is impaired when autophagy is activated by certain factors, such as rapamycin [11]; however, whether it is possible to promote wound healing by directly inhibiting autophagy has not been reported. In our preliminary study, it takes seven days to heal the wound in normal Balb/c mice with or without exposure to Baf A1, which suggested that Baf A1 has no effects on wound healing in normal mice. We also evaluated the effects of the autophagy inducer rapamycin and autophagy inhibitor Baf A1 on chronic refractory wound healing in hyperglycemic mice; data showed that rapamycin delayed wound healing which is consistent with previous description, whereas Baf A1 accelerates wound healing comparing with the control. Based on these results, we focused on refractory trauma caused by hyperglycemia rather than trauma in normal mice to investigate the mechanism of Baf A1 function. Briefly, in this study, we used db/db mice (BKS. Cg-Dock7^m+/+^Lepr^db^/JNju strain) as a type 2 diabetes mellitus (T2DM) model, established a full-thickness skin wound as a chronic refractory wound model, and then evaluated the effect of Baf A1 on wound healing. To investigate the underlying mechanism, we evaluated the effect of Baf A1 on cell proliferation, collagen production, and inflammatory cytokine secretion during wound healing to assess the relationship between autophagy and wound healing.

## 2. Materials and Methods

### 2.1. Experimental Animals

The db/db mice (BKS.Cg-Dock7^m+/+^Lepr^db^/JNju mice), development by gene mutations in leptin receptor, resulting in obesity, insulin resistance, hyperglycemia, fatty liver, refractory wound, and other symptoms, have been widely used for studies of T2DM due to their various characteristics related to T2DM [16, 17]. A total of 40 diabetic db/db and 20 nondiabetic db/m male mice were purchased from Changzhou Cavens Laboratory Animal, Ltd. The mice were 8–10 weeks old and were maintained under a 12-h light/dark cycle with unlimited access to food and water. All mice were fed in a single IVC cage, and the padding in the cage was changed every 2 days. All procedures were carried out in accordance with the National Institutes of Health Guide for the Care and Use of Laboratory Animals and were approved by the Committee on the Ethics of Animal Experiments at Xinxiang Medical University.

### 2.2. Model Preparation

Pentobarbital of 50 mg/kg concentration was injected intraperitoneally to anesthetize mice. Next, the dorsal hair was shaved, and the skin was sterilized with betadine and alcohol. Surgical scissors were used to make full-thickness skin defects (1.5 × 1.5 cm^2^) on the back of mice, and the wounds were stanched with sterile gauze. After the wound model was prepared, penicillin-streptomycin is used to prevent infection. All mice were fed in a single IVC cage, and the sterilized padding in the cage was changed every day.

### 2.3. Grouping

Blood glucose levels and weight changes were monitored every day, and mice with a blood glucose level >16.7 mmol/L were considered diabetic and were used in our study. The experiment was divided into three groups of 20 mice: db/m group (nondiabetic), db/db group (diabetic), and db/db treated with Baf A1 group. Baf A1 was dissolved in DMSO at 100 mg/mL, and diluted to 0.1 mg/mL with normal saline before intraperitoneal injection. Mice in Baf A1-treated group were treated with an intraperitoneal injection of Baf A1 solution at a dose of 1 mg/kg per day from the day of surgery until the mice were sacrificed, whereas mice in the remaining groups were administered an equivalent volume of saline. Three mice in each group were sacrificed at five sampling points (1, 3, 6, 9, and 14 days) for histological analysis. Anesthetized mice were euthanized with cervical dislocation. When mice never breathed after dislocation, euthanasia was scored as successful [18]. All methods were performed in accordance with the relevant guidelines and regulations. Every effort was made to minimize the number of animals used and their suffering. Blood glucose and body weight were also determined before sacrificing the mice. Blood glucose was measured with a glucometer (ACCU-CHEK; Roche), and body weight was measured with an electronic balance (Secura1102-1CN; Sartorius).

### 2.4. Blood and Serum Assays

Blood was collected into ethylene glycol tetra acetic acid-coated capillary blood collection tubes (BD Biosciences, 365973), and the complete blood count was determined with a blood analyzer (Forcyte Hematology Analyzer; Oxford Sciences, Oxford, CT). Commercial kits were used to assay serum levels of *β*-hydroxybutyrate (Shanghai MLBIO Biotechnology Co. Ltd., m1062014).

### 2.5. Wound Area Measurement

Wound images were obtained using a digital camera (Olympus E-M10 II) every day until the wounds closed completely. Morphometric analysis of the wounds was performed using images of the wounds acquired on days 0, 3, 6, 9, 14, and 21 postwounding to measure the remaining wound area using Photoshop (Adobe Photoshop CS5). The rate of wound closure was determined as the percent reduction from the original wound size and was calculated using the following formula: [(original wound area–current wound area)/original wound area] × 100%.

### 2.6. Histological Analysis

The tissue was collected from the wound area and fixed with 4% paraformaldehyde overnight. The tissue was then dehydrated in a graded series of alcohol and embedded in paraffin. The tissues were cut into 4 *μ*m sections for routine hematoxylin and eosin (H&E) and Masson's trichrome (Beyotime) staining to assess collagen formation. The stained sections were observed under a light microscope (NIKON Eclipse Ci, Japan) and digitally captured using a digital slice scanner (Pannoramic MIDI; 3D HISTECH, Hungary). An image analysis system (Quant center 2.1; 3D HISTECH, Hungary) was used to measure the length of the wound area, the thickness of the new epidermis, and collagen deposition. Five random fields from the central sections of each wound were obtained to exclude edge artifacts from histologic processing. The measurements were performed at 4× magnification.

### 2.7. Tissue Immunofluorescence Staining

Immunofluorescence was performed to detect LC3-II and P62, two autophagy-related markers. After deparaffinization and rehydration, antigen retrieval was performed in citric acid buffer (pH 6.0). The autophagosomes were detected by incubation with a rabbit anti LC3-II antibody (LC3A/B, 1 : 200, GB11124; Servicebio) and then stained with the secondary antibody (FITC-conjugated anti-rabbit antibody, 1 : 400, GB25303; Servicebio). The adaptor protein P62 was detected by incubation with a rabbit anti-P62 antibody (SQSTM1/p62, 1 : 400, 39786s, CST), and then with the secondary antibody (CY3-conjugated anti-rabbit antibody, 1 : 500, GB21303; Servicebio). The nuclei were counterstained with 4′,6′-diamidino-2-phenylindole (DAPI). The stained samples were imaged using fluorescent microscopy (NIKON Eclipse Ci, Japan). The Image Pro Plus 6.0 software (Media Cybernetics, Rockville, MD, USA) was used to measure the fluorescence intensity of the area per microscopic field occupied by LC3-II- or P62-positive staining at a magnification of 200× (*n* = 6 fields).

### 2.8. Tissue Immunohistochemical Staining

For immunohistochemistry, the wound tissues were deparaffinized, and endogenous peroxidase activity was blocked with 3% H_2_O_2_. All slides were pretreated with sodium citrate buffer or Tris/EDTA buffer for heat-mediated antigen retrieval. Next, the slides were incubated with 10% serum to block nonspecific binding. Tissue sections were labeled with primary antibodies against proliferating cell nuclear antigen (PCNA; 1 : 200, GB13010-1; Servicebio), tumor necrosis factor-*α* (TNF-*α*; 1 : 100, AB6671; ABCAM), or interleukin-10 (IL-10; 1 : 200, GB11534; Servicebio). After incubation with HRP-conjugated secondary antibodies, the sections were exposed to DAB to visualize the antigen signals and then counterstained with hematoxylin. The sections were viewed under a microscope, and images were obtained with a digital slice scanner (Pannoramic MIDI, 3D HISTECH, Hungary). The Image Pro Plus 6.0 software (Media Cybernetics, Rockville, MD, USA) was used to measure the density of the PCNA, TNF-*α*, or IL-10-positive staining area per microscopic field of view containing at a magnification of 200× (*n* = 6 fields).

### 2.9. Statistical Analysis

Significant differences between groups were determined using one-way or two-way ANOVA followed by Tukey's post hoc test for multiple comparisons. All data were obtained from at least three independent experiments. Significant differences between groups were determined using one-way or two-way ANOVA followed by Tukey's post hoc test for multiple comparisons. The statistical program used was GraphPad Prism 8 (San Diego, CA, USA). Data are expressed as the mean ± standard error of the mean (SEM). *P* < 0.05 was considered to indicate a statistically significant difference.

## 3. Results

### 3.1. Baf A1 Accelerates Wound Healing in db/db Mice

**Figure 1:**
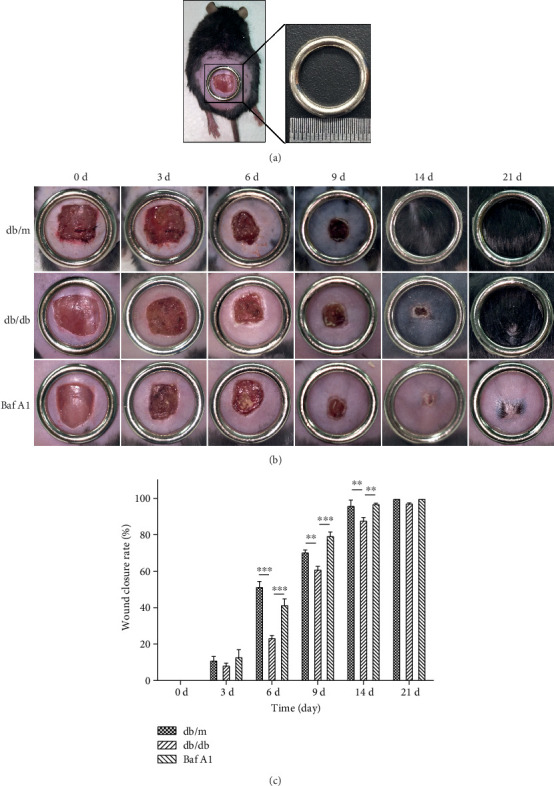
Repair of full-thickness wounds in diabetic (db/db) mice treated with and without Baf A1. (a) The wound healing model used in this study. (b) Representative images of healing wounds in db/m (normal control), db/db (diabetic), and Baf A1 (Baf A1-treated db/db) mice on days 0, 3, 6, 9, 14, and 21 postwounding. (c) Wound closure rates of the three groups at the indicated times. Data are presented as the mean ± SEM. ∗∗∗*P* < 0.001 and ∗∗*P* < 0.01. *n* = 3.

To determine whether Baf A1 enhances chronic refractory wound healing, a diabetes animal model with a full-thickness skin wound was created on the dorsal surface of db/m and db/db mice, and wound closure was monitored daily. Based on macroscopic observations, in the first three days, the wounds in the three groups gradually hardened and scabbed (Figures 1(a) and 1(b)). Starting on day 6, the wound healing rates were consistently lower for db/db mice than for db/m mice. More importantly, wound treatment with Baf A1 significantly accelerated wound closure (Figure 1(c)). These data suggested that Baf A1 accelerated skin wound healing in diabetic mice.

### 3.2. Histological Analysis of Wound Healing

**Figure 2:**
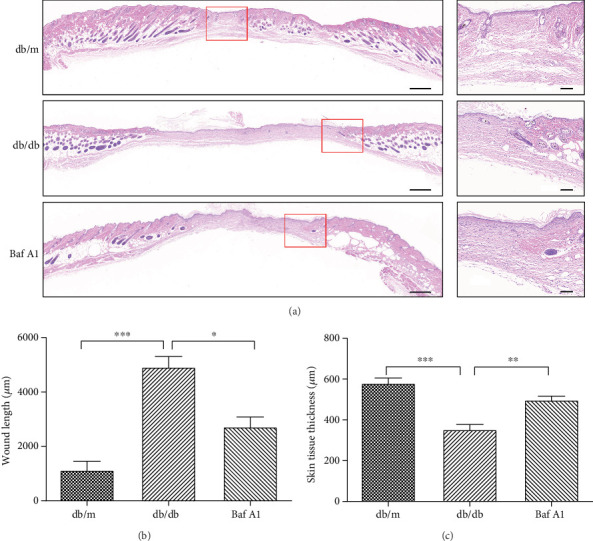
Histological analysis of wound healing. (a) Wound sections collected on day 14 postwounding were subjected to H&E staining. Scale bar = 500 *μ*m. The enlarged images on the right show the detailed structure of the new epidermis. Scale bar = 100 *μ*m. (b) Length of the wound area. (c) Thickness of the new epidermis. Data are presented as the mean ± SEM. ∗∗∗*P* < 0.001, ∗∗*P* < 0.01, and ∗*P* < 0.05. *n* = 3.

On day 14, three mice in each group were sacrificed, and the wound skin was harvested for histologic analysis. Representative images of H&E-stained skin sections collected on day 14 are shown in Figure 2. The length of the wound area in the Baf A1 group on day 14 was significantly shorter than that in the db/db group, although the db/m group (nondiabetic) showed superior wound healing (Figures 2(a) and 2(b)). As the enlarged images of H&E-stained tissues show, in the db/m and Baf A1-treated groups, a near-normal strata structure with a continued degree of epidermal hyperplasia was present, the reepithelialization process was rapid, and the basal layer, stratum spinosum, stratum granulosum, stratum lucidum, and stratum corneum could clearly be observed. In the db/db group, only the basal layer and stratum corneum could be observed (Figure 2(a)). Consistently, both the db/m group and Baf A1-treated group showed a significantly thicker epidermal layer than the db/db group (Figure 2(c)). Taken together, these data suggested that Baf A1 accelerated chronic refractory wound healing in diabetic mice and had a good healing effect.

### 3.3. Baf A1 Inhibits Autophagy Flux

**Figure 3:**
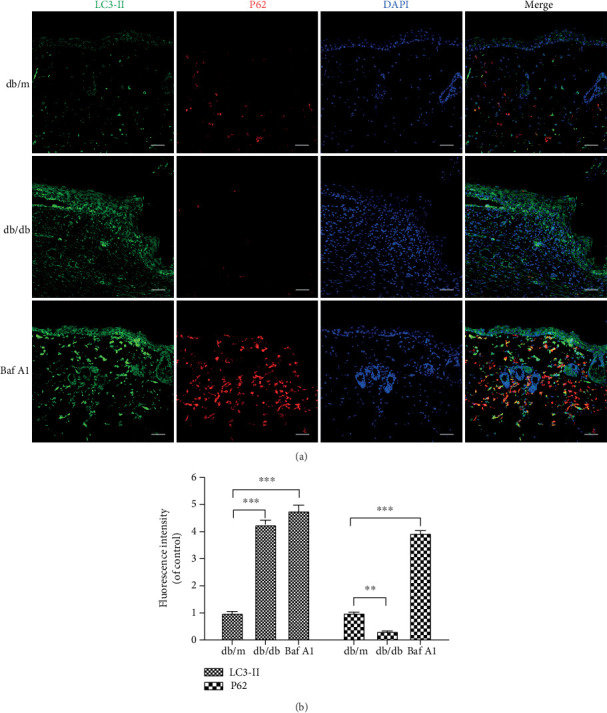
Effects of Baf A1 on autophagy. (a) Double immunofluorescence staining for the LC3-II (green) and P62 (red) protein in skin tissue samples obtained at 9 days postwounding. The nuclei were counterstained with DAPI (blue). Scale bar = 50 *μ*m. (b) Fluorescence intensity of the LC3-II (green) and P62 (red) protein in the db/db and Baf A1-treated groups compared to the db/m group. Data are presented as the mean ± SEM. ∗∗∗*P* < 0.001, and ∗∗*P* < 0.01. *n* = 3.

Microtubule-associated protein light chain 3 (LC3) is a marker of autophagy, and LC3-II levels are correlated with the number of autophagosomes. The adaptor protein P62 connects the LC3 protein to an ubiquitination substrate and can be incorporated into phagosomes via autophagic lysosomal degradation, marking the completion of autophagic flux; thus, the accumulation of P62 indicates disrupted autophagic degradation. In this study, LC3-II and P62 protein levels were assessed by immunofluorescence. The results showed that the intensity of LC3-II fluorescence was higher in the db/db and Baf A1-treated groups than in the db/m group. We also observed a significant reduction in P62 fluorescence intensity in the db/db group when compared with that in the db/m group; however, P62 fluorescence intensity in the Baf A1-treated group increased significantly (Figures 3(a) and 3(b)). The fluorescence intensity quantification showed that LC3-II increased and P62 expression decreased simultaneously in the db/db group when compared with the levels in the db/m group, indicating that autophagy flux is activated in the db/db group. In contrast, treatment with Baf A1 increased P62 protein levels compared with those in the db/m group, suggesting that the accumulation of LC3-II results from the inhibition of autophagic lysosomal degradation. In summary, autophagy flux is activated in db/db mice, and Baf A1 inhibits autophagy flux.

### 3.4. Baf A1 Has Minor Effects on Metabolism in db/db Mice

**Figure 4:**
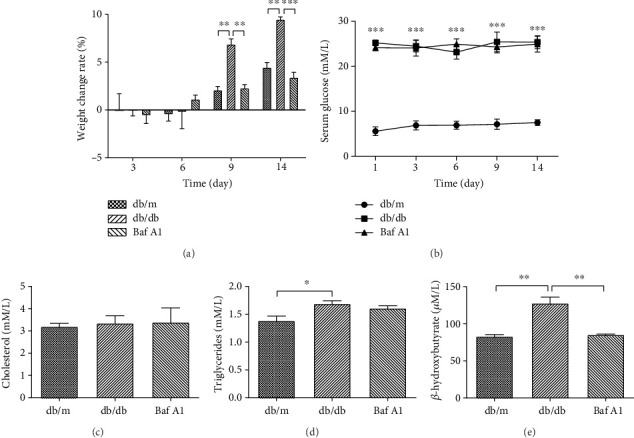
Metabolic effects of Baf A1 in db/db mice. (a) The rate of weight change at 3, 6, 9, and 14 days postwounding compared with the prewound weight. ∗∗∗*P* < 0.001 and ∗∗*P* < 0.01. (b) Serum glucose levels. ∗∗∗*P* < 0.001 vs. db/m. (c) Serum cholesterol levels on day 9. (d) Serum triglyceride levels on day 9. ∗*P* < 0.05. (e) Serum *β*-hydroxybutyrate levels on day 9. ∗∗*P* < 0.01. Data are presented as the mean ± SEM. *n* = 3.

Many findings have suggested that decreased autophagy may play a role in the dysregulation of the innate immune response in hyperglycemic mice. To examine this possibility, we examined the rate of weight change and serum glucose levels during wound healing, as well as cholesterol, triglyceride, and *β*-hydroxybutyrate serum levels at 9 days postwounding. During the first 6 days of wound healing, the mice in all three groups had the same rates of body weight change (Figure 4(a)). This resulted, in part, from decreased food intake. After 9 days, mice in the db/db group showed significant weight gain compared to mice in the db/m group, and the Baf A1-treated and db/m groups showed similar slightly increased weight change rates (Figure 4(a)). Compared with the db/m group, the blood glucose levels of the db/db and Baf A1-treated groups remained high throughout the experiment (Figure 4(b)). Mice in the three groups had equivalent cholesterol (Figure 4(c)) and triglyceride (Figure 4(d)) levels. The db/db group had increased rates of *β*-oxidation, as indicated by elevated serum levels of *β*-hydroxybutyrate (Figure 4(e)); this phenomenon is consistent with the characteristics of T2DM mice. Therefore, Baf A1 has minor effects on metabolism in db/db mice.

### 3.5. Baf A1 Promotes Cell Proliferation and Collagen Production

**Figure 5:**
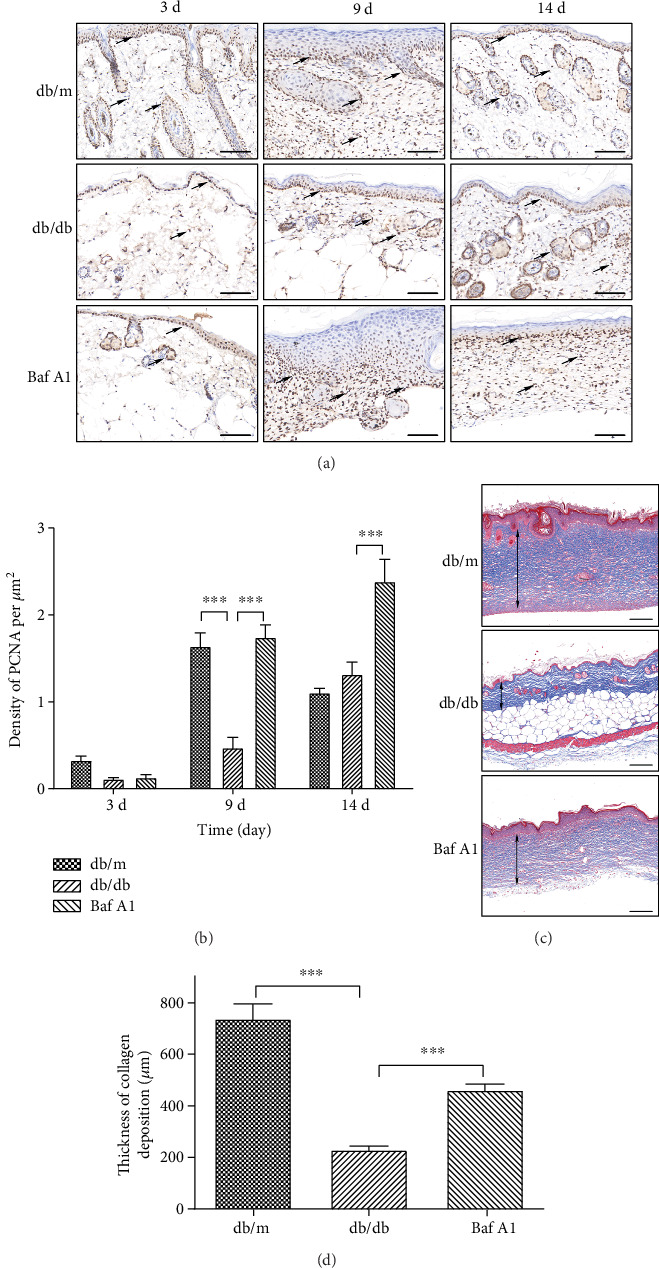
Effect of Baf A1 on cell proliferation and collagen production. (a) Representative images of immunohistochemical staining for PCNA taken at 3, 9, and 14 days postwounding; the black arrows indicate the PCNA-positive cells. Scale bar = 100 *μ*m. (b) Density analysis density of the PCNA-positive cells (per *μ*m^2^) in the newly generated epidermis. (c) Masson's trichrome staining was performed to measure collagen production at 14 days postwounding. Scale bar = 200 *μ*m. (d) Measurement of the thickness of the collagen deposition from the dermal-subcutaneous interface to the external surface of the epidermis. Data are presented as the mean ± SEM. ∗∗∗*P* < 0.001. *n* = 3.

As Baf A1 promoted wound healing and showed good healing effects in the diabetic mice (Figures 1 and 2), we investigated the underlying mechanism. To this end, the PCNA protein in the skin was detected by immunohistochemistry at 3, 9, and 14 days postwounding. On day 3, we observed PCNA-positive cells mainly concentrated at the wound edge, and the density of PCNA-positive cells in the db/m group was slightly higher, but the difference was not significant. On day 9, the density of PCNA-positive cells was significantly higher in the db/m and Baf A1-treated groups than in the db/db group. On day 14, the density of PCNA-positive cells increased slightly in the db/db group but was still much lower than that in the db/m group and Baf A1-treated groups (Figure 5(a)). Moreover, the statistical analysis showed that the density of PCNA-positive cells per field was higher in the db/m and Baf A1 groups than in the db/db group (Figure 5(b)), suggesting that Baf A1 could regulate cell proliferation resulting in epidermal hyperproliferation.

We also evaluated the extent of fibrosis using Masson's trichrome staining on samples obtained at 14 days postwounding, and the thickness of collagen deposition was measured from the dermal-subcutaneous interface to the external surface of the epidermis. Representative images are shown in Figure 5(c), and a summary of the measurements is shown in Figure 5(d). In the db/m group and the Baf A1 treatment group, collagen filling is relatively sufficient, while in the db/db group, a large number of hollow areas lack collagen filling. The collagen deposition in the newly generated skin of mice in the db/m group was much thicker than that in the db/db group. Likewise, the collagen deposition in the Baf A1-treated group was thicker than that in the db/db group. These results suggest that Baf A1 promotes cell proliferation and collagen production.

### 3.6. Baf A1 Regulates Inflammatory Balance

**Figure 6:**
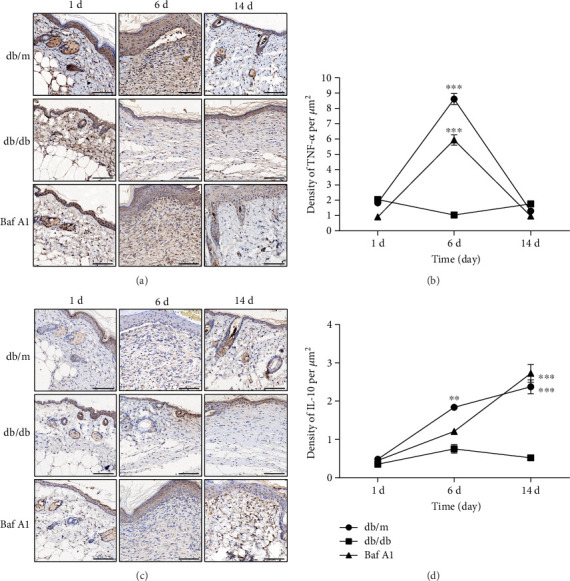
Effect of Baf A1 on the expression of the proinflammatory cytokine TNF-*α* and the anti-inflammatory cytokine IL-10. (a) Representative images of immunohistochemical staining for TNF-*α* at 1, 6, and 14 days postwounding. Scale bar = 100 *μ*m. (b) Density analysis of TNF-*α*-positive cells per *μ*m^2^ in the newly generated epidermis. (c) Representative images of immunohistochemical staining for IL-10 at 1, 6, and 14 days postwounding. Scale bar = 100 *μ*m. (d) Analysis of the density of IL-10-positive cells per *μ*m^2^ in the newly generated epidermis. Data are presented as the mean ± SEM. ∗∗∗*P* < 0.001 vs. db/db and ∗∗*P* < 0.01 vs. db/db. *n* = 3.

To investigate the effect of Baf A1 on inflammation, we analyzed the proinflammatory cytokine TNF-*α* and the anti-inflammatory cytokine IL-10 in the subcutaneous areas of the wound edge at 1, 6, and 14 days postwounding. On day 6, a marked increase in the expression level of the proinflammatory cytokine TNF-*α* was observed in the wound area of mice in the db/m and Baf A1-treated groups. Then, on day 14, the TNF-*α* level was reduced to that on day 1. In contrast, there was no significant change in the db/db group (Figure 6(a)). The statistical analysis result revealed that TNF-*α* expression peaked on day 6 in the db/m and Baf A1-treated groups, while the level in the db/db group was unchanged (Figure 6(b)). For the anti-inflammatory cytokine IL-10, a slight increase at the wound edge was observed in all three groups on day 6 when compared to that on day 1. On day 14, IL-10 expression increased significantly in the db/m and Baf A1-treated groups; however, IL-10 expression in the db/db group was mainly at the wound edge, and the level was unchanged (Figure 6(c)). Statistical analysis also showed that IL-10 expression in the db/m and Baf A1-treated groups was significantly increased after 6 days, while that in db/db group showed no significant change (Figure 6(d)). These results revealed defects in the regulation of the inflammatory balance in db/db mice and showed that Baf A1 could activate inflammation in the early phase of the wound healing process and improve the ability of db/db mice to regulate the inflammatory balance.

## 4. Discussion

Autophagy is an evolutionarily conserved degradation process related to homeostasis that eliminates intracellular proteins and damaged organelles, and it has recently emerged as a potent, clinically relevant modulator of disease progression [10]. Some studies have suggested that enhancement of autophagy negatively impacts wound healing and diabetic wounds. However, there is not enough evidence to show whether attenuation of autophagy positively impacts chronic refractory wounds, and the underlying molecular basis is also unclear. In the current study, we used a db/db mouse wound healing model and employed several approaches to address these questions. The major finding of this study is that inhibiting autophagy by administering Baf A1 positively impacts chronic refractory wounds in db/db mice by promoting cell proliferation and collagen production and modulating the inflammatory balance, which was supported by the following findings: [1] Baf A1 accelerates chronic refractory wound healing in diabetic mice and has a good healing effect, [2] Baf A1 inhibits autophagy flux in db/db mice, [3] Baf A1 has minor effects on metabolism in db/db mice, [4] Baf A1 promotes cell proliferation and collagen production, and [5] Baf A1 regulates the inflammatory balance.

In our study, the tissue from the chronic lesions of diabetic mice, a typical model of impaired wound healing, also appeared to have an increased level of autophagy flux, accompanied by increased LC3-II levels and decreased p62 levels. These results are consistent with several clinic studies, which showed that the major side effect of sirolimus, an immunosuppressive agent administered for antirejection of graft after surgery, was delayed wound closure [19, 20]. One possibility is that the drug activated the autophagic pathway, since sirolimus is a specific inhibitor of mTOR and an autophagy inducer. Our previous study had similar results. In this work, we showed the specific autophagy inhibitor Baf A1 restored delayed cutaneous healing. The diabetic model mice showed delayed wound healing when compared with nondiabetic mice. However, following the administration of Baf A1, no significant differences were observed between db/m and Baf A1-treated db/db mice, and histological analysis also showed a better healing effect in the Baf A1-treated group than in the db/db group. These results are consistent with our hypothesis that attenuation of autophagy has a positive impact on chronic refractory wounds. In addition, there are some interesting clues in our results, for example, diabetic mice are prone to keloid scar formation and the hair growth in mice was inhibited by Baf A1 treatment (Figure 1). This indicated that autophagy may be closely related to the formation of keloid scar and skin appendages such as sweat glands and hair follicles. Our subsequent work may involve this topic.

In most cell types, autophagy activity is at basal levels and plays a housekeeping role, maintaining normal cellular functions, and both reduced and excessive autophagy can cause various diseases. It has been reported that the insufficient podocyte autophagy was involved in the pathogenesis of exacerbated proteinuria in diabetic nephropathy [21]. In contrast, excessive autophagy in adipocytes caused insulin resistance in the adipose tissue of obese patients with type 2 diabetes [22]. In our study, excess autophagy caused delayed wound healing in db/db mice. However, when the db/db mice were treated with Baf A1, the increased levels of LC3-II and p62, and the wound healing indicated that inhibition of autophagic flux by Baf A1 significantly counteracted the damage caused by activation of autophagy in db/db mice. It is known that Baf A1 impairs autophagic flux by preventing the fusion of autophagosomes and lysosomes because Baf A1 is a macrolide antibiotic that inhibits the acid pumping functions of the vacuolar H^+^ ATPase with high specificity and at nanomolar concentrations [23, 24]. Baf A1 disrupts the functions of multiple acidic organelles within the central vacuolar system of the cell [25]. Thus, the wound healing-promoting effects induced by Baf A1 in db/db mice are mainly mediated by inhibition of autophagic flux. Baf A1 also has minor effects on metabolism in db/db mice; therefore, promotion of wound healing was not secondary to the metabolic effect induced by Baf A1 treatment.

Wound healing is a complex biological process involving many cell types. Fibroblasts and keratinocytes in the skin participate in complex interactions to maintain homeostasis in the skin and are critical during wound healing [26, 27]. To further investigate the underlying mechanism, the PCNA protein, which is a marker of cell proliferation, was detected by immunohistochemistry, and collagen deposition was measured by Masson's trichrome staining. We observed that Baf A1 enhanced PCNA expression in keratinocytes when compared with the levels in untreated db/db mice, suggesting that the keratinocytes in Baf A1-treated db/db mice were hyperproliferating. This was consistent with a previous study showing that active keratinocyte proliferation is important for new tissue regeneration [28–30]. It is difficult to explain how Baf A1 promotes proliferation of keratinocyte, since current research generally suggests that a degree of autophagy is necessary for keratinocyte proliferation [31, 32]. Research by Lee et al. have shown that keratinocyte autophagy negatively regulates p62 expression, and genetic knockdown of p62 reduces the production of inflammatory cytokines and cell proliferation [33]; this result could partly explain why Baf A1 can promote the proliferation of keratinocytes.

Collagen deposition in the Baf A1-treated group was thicker than that in the db/db group, which was also consistent with reports that fibroblasts proliferation and collagen synthesis aid in wound healing by contracting the wound [26, 34]. The type I and III collagens are primary constituents in dermal extracellular matrix (ECM), which is very important in wound healing [35]. This is an important aspect of wound healing as early secretion of collagen type III helps in accelerated repair process; however, persistent or higher deposition of collagen type III is associated with scar formation during wound healing [36]. So, the formation of collagen type may be closely related to autophagy and thus influence the formation of keloid scar. Therefore, it is of great significance to further study the effect of autophagy on the formation of collagen type.

The wound healing process has three phases: inflammation, new tissue regeneration (proliferation), and remodeling (maturation). The inflammation process is the phase most affected by diabetes, whereas inflammation is the initial, key step in wound healing. A proper inflammatory response is important for wound repair, as too little inflammation could lead to unhealed tissue [37], whereas exacerbated and prolonged inflammation impairs wound healing and increases scarring [38–40]. Therefore, the effect of Baf A1 on inflammation in vivo was also assessed. We showed that the expression of the proinflammatory cytokine TNF-*α* in db/db mice remained largely unchanged during healing; after treatment with Baf A1, TNF-*α* expression peaked on day 6, which is similar to the db/m group. In contrast, the expression of the anti-inflammatory cytokine IL-10 increased slightly in all three groups on day 6 and increased significantly in the db/m and Baf A1-treated groups but remained unchanged in the db/db group on day 9. The inflammatory response is a fundamental response to harmful stimuli, such as disease and injury, and is indispensable for wound healing [41]. Our findings showed that the outcome of delayed healing in db/db mice was due to the lack of a proper inflammatory response at the early phase, and Baf A1 activated inflammation and improved the ability of db/db mice to regulate the inflammatory balance. Similar to our findings, impaired macrophage autophagy was shown to increase the immune response in obese mice by promoting proinflammatory macrophage polarization [42]. In addition, delayed healing due to impaired autophagy also resulted in a large population of M1 macrophages and sustained inflammation in mice [11]. These discrepancies may be due to the various cell types studied and different pathological situations. In our opinion, the inflammatory response is affected by the level of autophagic flux, which is important to the process of wound healing. In this study, the inhibition of autophagic flux by Baf A1 is a major reason to accelerate chronic refractory wound healing in db/db mice. There are many other factors that can influence the inflammatory response, for example, both hyaluronidases and metalloproteinases have been reported to regulate the inflammatory response by pro- and anti-inflammatory cytokines during wound healing [43, 44]; so, whether Baf A1 regulates inflammation levels by affecting hyaluronidases and metalloproteinases activity is an interesting hypothesis. The detailed molecular mechanisms underlying Baf A1-mediated regulation of the inflammatory balance will be studied in the future.

## 5. Conclusion

In summary, we determined the effect of Baf A1, an inhibitor of autophagic flux, on chronic refractory wound healing. The mechanism underlying the accelerated wound healing induced by Baf A1 is believed to involve inhibition of autophagic flux. Further investigation showed that Baf A1 promotes cell proliferation and collagen production and regulates the inflammatory balance during healing. These results indicate that Baf A1 may be a promising candidate for wound therapy, although additional work is needed to elucidate the underlying mechanisms before treating chronic refractory wounds.

## Data Availability

The data used to support the findings of this study are available from the corresponding author upon request.
